# Supplementary material for: Outpatient Antibiotic Prescriptions in France: Patients and Providers Characteristics and Impact of the COVID-19 Pandemic
Source: Antibiotics (Basel). 2022 May 11;11(5):643. doi: 10.3390/antibiotics11050643 (PMC9137595; doi:10.3390/antibiotics11050643)
Supplement: Supplementary file 1 [file antibiotics-11-00643-s001.zip › antibiotics-1697020-supplementary.pdf]

Supplementary Materials

Description of the database and variables

Database

The SNDS (*Système National des Données de Santé* – French national health data system) is a medical-administrative data warehouse covering all health insurance schemes and almost all of the French population. It is built from a prospective, individualised and exhaustive collection of all care services presented to reimbursement. Only the use of drugs by patients residing in healthcare facilities with an internal pharmacy does not appear in the SNDS. The data is recorded in the form of individual data with treatment dates of care and detailed coding of the drugs and services provided. Pseudonymised identifiers link the patients' individual information with all outpatient services (*Datamart de consommation inter-régimes* [DCIR]) as well as with data from the programme for the medicalisation of information systems (PMSI) during hospitalisation in public or private facilities (1). Similarly, the administrative information such as age, gender, specialty, type of practice, conventional status, etc. of the health professionals associated with each of the outpatient services presented to reimbursement is available in a repository.

Variables

Long-term illnesses (LTI) are serious and/or chronic illnesses defined by a list of conditions established by decree and which entitle the insured to exemption from co-payment (100% reimbursement) for care related to these illnesses. A patient is classified as suffering from a long-term illness when he/she benefits from the exemption system for long-term illnesses, i.e. when at least one of his or her benefits is fully reimbursed as an exemption from co-payment for a long-term illness during the study period. Thus, the number of cumulative long-term conditions per patient is estimated and classified as follows: 0, 1 and ≥ 2.

Complementary universal health insurance (CMUc): it is used as an indicator of precariousness since its allocation is based on individual resources. However, this indicator cannot be used after the age of 60 since the amount of the minimum old-age pension paid to persons with low resources exceeds the limit for receiving CMUc.

French deprivation index (Fdep): it is calculated at municipal district level and helps to establish the socio-economic background of recipients on the basis of several parameters (median household income, proportion of higher education graduates, employment and unemployment within the district). The municipal districts are then stratified into population quintiles according to the level of disadvantage of the population, ranging from the least disadvantaged quintile (Q1) to the most disadvantaged quintile (Q5).

Urban area: these are densely populated residential areas (1500 inhabitants/km^2 and ≥ 50,000 inhabitants) and of intermediate density (300 inhabitants/km^2 and ≥ 5000 inhabitants).

Rural area: these are less dense residential areas (300 inhabitants/km² and 5000 inhabitants) or of very low density (25 inhabitants/km² and 300 inhabitants).

Healthcare professional's (HCP) type of practice: this is the legal status under which the HCP works. HCPs are referred to as private or salaried when they carry out all activities under this status, regardless of the location (hospital, outpatient setting). When HCPs carry out both private and salaried work this is referred to as mixed practice.

Affiliation with state healthcare system: this determines the prices applied by HCPs. While sector 2 allows the HCP to freely set the price for their services, sector 1 does not allow nationally regulated fees to be exceeded.

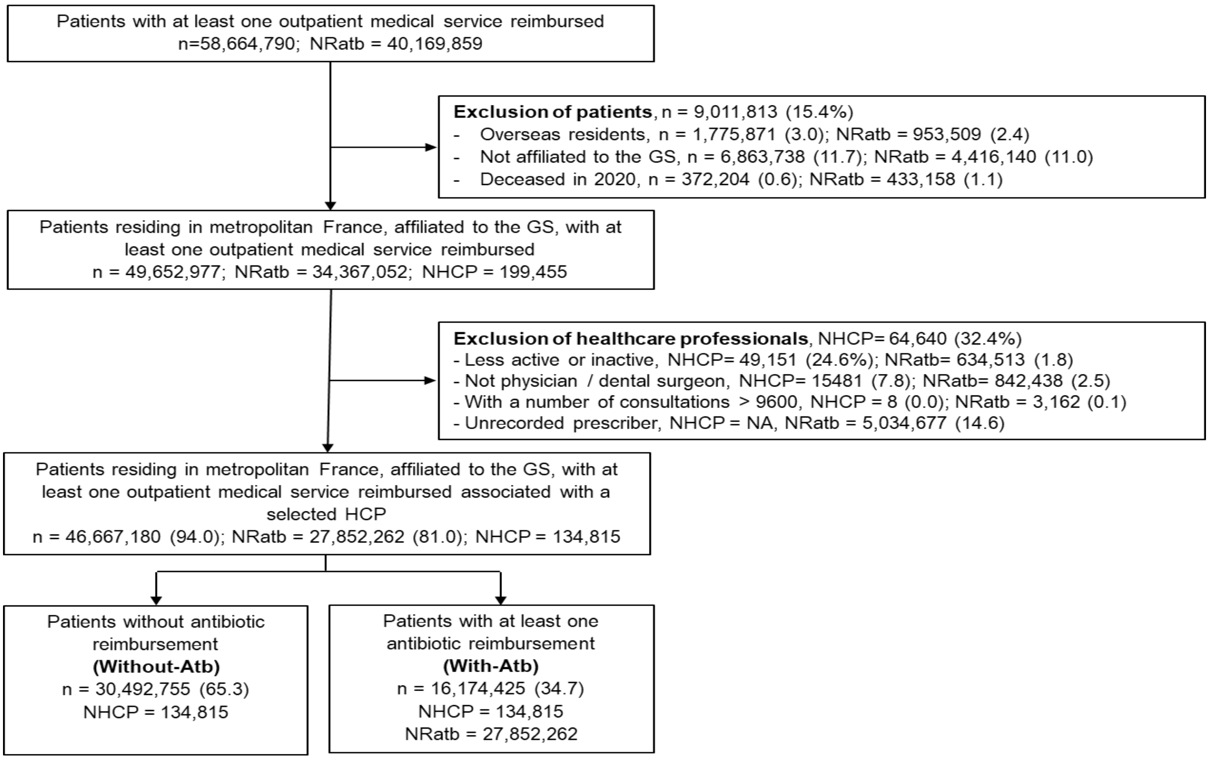

Figure S1: Flowchart of patient and prescriber selection from French health insurance databases (SNDS) - 2020.
Key:
n: Number of patients
NRatb: Number of reimbursements for antibiotics
NHCP: Number of healthcare professionals
HCP: Healthcare professional
GS: General scheme (main public health insurance scheme in France)

Bibliography

1. Tuppin P, Rudant J, Constantinou P, Gastaldi-Ménager C, Rachas A, de Roquefeuil L, et al. Value of a national administrative database to guide public decisions: From the système national d'information interrégimes de l'Assurance Maladie (SNIIRAM) to the système national des données de santé (SNDS) in France. Rev Epidemiol Sante Publique. 2017 Oct;65 Suppl 4:S149–67.

Table S1: Socio-demographic characteristics and distribution of prescriptions for children (<15 years old) with at least one antibiotic prescription reimbursement (With-Atb), 2019 and 2020, SNDS.

	**With-Atb 2019** **N =** 3,501,798 (41.2)	**With-Atb 2020** **N=** 2,624,533 (31.2)	**Delta 2019-2020** **N=** 877,265 (25.1)
**Socio-demographic characteristics**			
Sex, n (%)			
Boy	1,787,349 (51.0)	1,341,525 (51.1)	445,824 (24.9)
Girl	1,714,449 (49.0)	1,283,008 (48.9)	431,441 (25.2)
Age, in years, n (%)			
[0 - 5[	1,528,973 (43.7)	1,133,255 (43.2)	395,718 (25.9)
[5 - 15[	1,972,825 (56.3)	1,491,278 (56.8)	481,547 (24.4)
Comorbidities, n (%)			
Chronic respiratory	253,410 (7.8)	197,970 (8.1)	55,440 (21.9)
**CMU-c** [1]			
Yes, n (%)	636,450 (18.2)	550,485 (21.0)	85,965 (13.5)
**Use of care**			
**Number of consultations, *n (%)***			
[1 - 2[	525,860 (15.0)	717,778 (27.4)	-191,918 (36.5)
[3 - 4[	861,249 (24.6)	795,436 (30.3)	65,813 (7.6)
[5 - 9[	1,444,141 (41.2)	864,821 (33.0)	579,320 (40.1)
[10 and +[	670.548 (19.2)	246,498 (9.4)	424,050 (63.2)
**Prescription per recipient, *n (%)***			
1	1,996,763 (57.0)	1,729,601 (65.9)	267,162 (13.4)
2	802,948 (22.9)	551,662 (21.0)	251,286 (31.3)
3	355,591 (10.2)	197,946 (7.5)	157,645 (44.3)
4 or more	346,496 (9.9)	145,324 (5.5)	201,172 (58.1)
**Antibiotic prescription**	**6,446,682**	**4,141,785**	**↓ 2,304,901 (35.8)**
**Therapeutic group**			
J01A Tetracyclines	59.521 (0.9)	59,269 (1.4)	252 (0.4)
J01C Penicillins	4,669,504 (72.4)	2,976,075 (71.9)	1,693,429 (36.3)
J01CA Penicillins with extended spectrum	3,875,950 (60.1)	2,423,606 (58.5)	1,452,344 (37.5)
J01CR Combinations of penicillins	782,662 (12.1)	544,766 (13.2)	237,896 (30.4)
J01D Other beta-lactams	1,046,980 (16.2)	640,281 (15.5)	406,699 (38.8)
J01DD 3G cephalosporins	973,377 (15.1)	601,339 (14.5)	372,038 (38.2)
J01E Sulphonamides and trimethoprim	57,874 (0.9)	43,689 (1.1)	14,185 (24.5)
J01F Macrolides	548,106 (8.5)	370,554 (9.0)	177,552 (32.4)
J01FA10: Azithromycin	161,335 (2.5)	137,015 (3.3)	24,320 (15.1)
J01M Quinolones	3,270 (0.1)	2,862 (0.1)	408 (12.5)
J01R Combinations of antibacterials[1]	21,712 (0.3)	17,546 (0.4)	4,166 (19.2)
J01X Other antibacterials	39,469 (0.6)	31,335 (0.8)	8,134 (20.6)

[1] Complementary universal health insurance (CMUc)

Table S2: Distribution of therapeutic classes of antibiotics dispensed according to the prescriber's subspecialty, 2019, SNDS.

	**GPs**[1] N= 26,566,106 (78.0)	**Dental surgeons** N=4,164,192 (12.2)	**Paediatricians** N= 742,006 (2.2)	**Dermatologists** N= 563,016 (1.7)	**Other subspecialties** N= 2,012,017 (5.9)
	**n (%)**	**n (%)**	**n (%)**	**n (%)**	**n (%)**
J01A Tetracyclines	594,014 (2.2)	4,079 (0.1)	216 (0.3)	466,558 (82.9)	65,232 (3.2)
J01C Penicillins	13,828,490 (52.0)	2,791,142 (67.0)	568,656 (76.6)	37,705 (6.7)	775,348 (38.5)
J01CA Penicillins with extended spectrum	10,290,861 (38.7)	2,331,766 (56.0)	454,836 (61.3)	14,029 (2.5)	38,382 (19.1)
J01CR Combinations of penicillins	3,340,219 (12.6)	458,944 (11.0)	112,667 (15.2)	17,310 (3.1)	377,818 (18.8)
J01D Other beta-lactams	3,169,494 (11.9)	7,368 (0.2)	83,940 (11.3)	2,160 (0.4)	162,645 (8.1)
J01DC 2G cephalosporins	477,753 (1.8)	1,354 (0.0)	4,112 (0.6)	348 (0.1)	24,299 (1.2)
J01DD 3G cephalosporins	2,679,665 (10.1)	5,814 (0.1)	79,769 (10.8)	1,707 (0.3)	137,271 (6.8)
J01E Sulphonamides and trimethoprim	317,182 (1.2)	871 (0.0)	8,548 (1.1)	1,119 (0.2)	48,065 (2.4)
J01F Macrolides	4,406,077 (16.6)	28,8341 (6.9)	70,542 (9.5)	39,718 (7.1)	305,496 (15.2)
J01M Quinolones	1,282,310 (4.8)	4,117 (0.1)	997 (0.1)	2,302 (0.4)	218,343 (10.5)
J01R Combinations of antibacterials[2]	653,691 (2.4)	1,061,386 (25.5)	3,565 (0.5)	3,220 (0.5)	213,283 (10.6)
J01X Combinations, antibacterials	2,314,018 (8.77)	5,403 (0.1)	3,546 (0.4)	10,018 (1.8)	217,709 (10.8)

[1] General practitioners; [2] Spiramycin and metronidazole

Table S3: Distribution of therapeutic classes of antibiotics dispensed according to the prescriber's subspecialty, 2020, SNDS.

	**GPs**[1] N= 20,786,542 (74.6)	**Dental surgeons** N= 4,208,530 (15.1)	**Paediatricians** N= 486,840 (1.8)	**Dermatologists** N= 560,958 (2.0)	**Other specialisms** N= 1,809,392 (6.5)
	**n (%)**	**n (%)**	**n (%)**	**n (%)**	**n (%)**
J01A Tetracyclines	592,645 (2.9)	3,683 (0.1)	2,330 (0.5)	465,843 (83.0)	62,237 (3.4)
J01C Penicillins	9,828,988 (47.3)	2,918,249 (69.3)	361,105 (74.2)	36,177 (6.5)	676,107 (37.4)
J01CA Penicillins with extended spectrum	6,863,715 (33.0)	2,421,536 (57.5)	284,013 (58.3)	12,013 (2.1)	315,946 (17.5)
J01CR Combinations of penicillins	2,793,342 (13.4)	496,351 (11.8)	76,285 (15.7)	18,581 (3.3)	347,025 (19.2)
J01D Other beta-lactams	2,188,488 (10.5)	5,745 (0.1)	54,775 (11.3)	1,624 (0.3)	127,300 (7.0)
J01DC 2G cephalosporins	252,495 (1.2)	929 (0.0)	2,361 (0.5)	202 (0.0)	16,023 (0.9)
J01DD 3G cephalosporins	1,929,430 (9.3)	4,656 (0.1)	52,382 (10.8)	1,293 (0.2)	110,247 (6.1)
J01E Sulphonamides and trimethoprim	327,469 (1.6)	799 (0.0)	6,873 (1.4)	1,209 (0.2)	51,311 (2.8)
J01F Macrolides	3,691,466 (17.8)	289,773 (6.9)	54,201 (11.1)	42,164 (7.5)	294,147 (16.3)
J01M Quinolones	1,136,150 (5.5)	3,447 (0.1)	990 (0.2)	1,961 (0.4)	197,268 (10.9)
J01R Combinations of antibacterials[2]	733,900 (3.5)	980,070 (23.3)	3,018 (0.7)	2,874 (0.5)	189,026 (10.4)
J01X Other antibacterials	2,280,084 (11.0)	5,358 (0.1)	3,259 (0.7)	8,904 (1.6)	208,182 (11.5)

[1] General practitioners; [2] Spiramycin and metronidazole

Table S4: Socio-demographic characteristics of antibiotic prescribers, overall and according to subspecialty, 2020, SNDS.

	All HCPs[1]		GPs[2]		Dentists		Paediatricians		Dermatologists		Other	
	n	%	n	%	n	%	n	%	n	%	n	%
Sex												
Women	55,190	41.1	23,867	41.9	15,664	43.9	1,770	67.5	1,892	69.6	11,997	33.2
Men	79,001	58.9	33,152	58.1	20,061	56.2	851	32.5	826	30.4	24,111	66.8
Age, in years												
< 50	54,583	40.9	22,000	38.9	16,671	46.4	963	37.5	675	25.2	11,700	32.8
≥ 50	78,866	59.1	34,607	61.1	19,245	53.6	1,606	62.5	2,004	74.8	23,978	67.2
Mean ± SD[3]	51.6 ± 12.5	-	52.3 ± 12.2	-	47.0 ± 13.2	-	53.5 ± 11.6	-	56.1 ± 10.8	-	54.4 ± 11.2	-
**Years of experience**												
Junior ≤15 years	54,034	40.9	22,333	40.0	14,744	41.9	1,292	50.7	754	28.4	14,911	41.7
Intermediate 15-30 years	45,801	34.7	19,457	34.8	11,782	33.4	733	28.8	1,048	39.4	12,781	35.8
Senior ≥ 30 years	32,210	24.4	14,065	25.2	8,705	24.7	524	20.6	858	32.3	8,058	22.5
Mean ± SD	19.0 ± 12.6	-	19.3 ± 12.9	-	18.9 ± 12.7	-	16.6 ± 12.8	-	22.4 ± 11.9	-	18.6 ± 12.2	-
**Type of practice**												
Private	126,018	93.5	54,144	94.8	34,783	96.4	2,255	85.8	2,422	88.8	32,414	89.4
Mixed	8,675	6.4	2,871	5.0	1,293	3.6	373	14.2	307	11.2	3,831	10.6
Salaried	122	0.1	119	0.2	1	0.0	0	0.0	0	0.0	2	0.0
**Conventional status**												
Sector 1	108,663	81.1	53,258	94.0	35,980	100.0	1,430	54.5	1,498	55.4	16,497	45.7
Sector 2	25,381	18.9	3,377	6.0	0	0.0	1,192	45.5	1,206	44.6	19,606	54.3
**Activity**												
Consultations	229,491,992	-	178,368,176	77.7	8,727,061	3.8	5,143,727	2.2	3,905,140	1.7	33,347,888	14.5
Patients seen	96,726,501	-	64,789,877	67.0	7,993,011	8.3	2,237,076	2.3	3,090,882	3.2	18,615,655	19.4
Prescriptions[4]	27,852,262	-	20,786,542	74.6	4,208,530	15.1	486,840	1.7	560,958	2.0	1,809,392	6.5
Prescription per 1000 consultations	121	-	117	-	-	-	95	-	144	-	54	-
**All Providers**	**134,815**	**-**	**57,134**	**42.4**	**36,077**	**26.8**	**2,628**	**2.0**	**2,729**	**2.0**	**36,247**	**26.9**

[1] healthcare professionals; [2] General practitioners; [3] Standard Deviation; [4] Antibiotic prescription
